# Early Life Inflammation and Neurodevelopmental Outcome in Bangladeshi Infants Growing Up in Adversity

**DOI:** 10.4269/ajtmh.17-0083

**Published:** 2017-07-17

**Authors:** Nona M. Jiang, Fahmida Tofail, Jennie Z. Ma, Rashidul Haque, Beth Kirkpatrick, Charles A. Nelson, William A. Petri

**Affiliations:** 1 Division of Infectious Diseases and International Health, Department of Medicine, School of Medicine, University of Virginia, Charlottesville, Virginia;; 2 International Centre for Diarrhoeal Disease Research, Bangladesh, Dhaka, Bangladesh;; 3 Division of Biostatistics, Department of Public Health Sciences, School of Medicine, University of Virginia, Charlottesville, Virginia;; 4 Department of Medicine, University of Vermont, Burlington, Vermont;; 5 Division of Developmental Medicine, Boston Children’s Hospital, Harvard Medical School, Boston, Massachusetts

## Abstract

Exposure to profound adversity can negatively affect the neurodevelopment of children, but biologic mechanisms that underlie this association remain unknown. We sought to determine whether elevated levels of the inflammatory markers C-reactive protein (CRP) and soluble CD14 (sCD14) are associated with neurodevelopmental outcomes in Bangladeshi children. A total of 422 infant–mother pairs from an urban slum in Dhaka, Bangladesh were enrolled at birth and followed prospectively. Inflammation was measured with sCD14, interleukin (IL)-1β, and IL-6 at 18 weeks, and CRP at 6, 18, 40, and 53 weeks. Psychologists assessed cognitive, language, motor, and social emotional development using the Bayley Scales of Infant and Toddler Development at 78 and 104 weeks of age. We tested for the association of inflammatory markers with developmental outcomes, independent of previously identified associations such as malnutrition, family income, and maternal education. Every 10 pg/mL increase in sCD14 was associated with a 1.1–2.0 decrement in cognitive and motor scores at 78 weeks and in all domains at 104 weeks. The cumulative number of CRP elevations that a child experienced in the first year of life, as well as IL-1β and IL-6 at 18 weeks of age, were also negatively associated with Bayley Scales results. CRP, sCD14, IL-1β, and IL-6 were associated with lower neurodevelopmental outcomes. Our findings implicate a role of inflammation in the neurodevelopment of children growing up in adversity.

## INTRODUCTION

Hundreds of millions of children growing up in poverty in low- and middle-income countries do not meet their full developmental potentials, which in turn affects their academic performance and future earnings.^1–3^ The identification of biomarkers that are associated with poor neurodevelopmental outcomes offers a promising approach to shed light on biological mechanisms that underpin the association between early adversity and development. In recent years, there has been a particular interest in the association of inflammatory markers with neurodevelopmental outcomes.^4–6^ The basis behind studying inflammatory markers is rooted in the idea that inflammation is known to be detrimental to the developing brain as shown in both animal and human studies.^7–9^

Our group has recently identified for the first time a connection of elevated inflammatory markers in children in low-income countries with neurodevelopmental outcomes. We found that children from an urban slum in Bangladesh with higher levels of pro-inflammatory cytokines had lower neurodevelopmental scores.^6^ It, however, remains unknown what is driving cytokine production in these children. Enteric infections remain a leading cause of morbidity in young children worldwide who carry a disproportionately large share of the disease burden.^10^ In this particular cohort of children, enteric infections are pervasive with children having an average of two different enteropathogens in their stool by 6 weeks of age.^11^ As such, inflammation triggered by recurrent enteric infections early in life may be the driving force behind elevations in systemic inflammatory markers in these children.

One important complication of enteric infections, either with or without overt diarrhea, is environmental enteropathy (EE), a condition characterized by gut inflammation, barrier disruption, and systemic inflammation.^12–14^ EE is thought to be caused by repeated enteric infections that results in blunting of the villi and microbial translocation.^12^ EE has been linked to poor health outcomes in children including oral vaccine failure and growth faltering.^13,15^ Although downstream effects of EE, such as systemic inflammation, have been linked to poor child development, the association between EE and neurodevelopment remains unknown.

Soluble CD14 (sCD14) is an early marker of immune activation during primary infection of the gut and may serve as a measure of gut barrier dysfunction.^13,16,17^ Thus, sCD14 can serve as a useful surrogate marker for EE.^13^ CD14 is found primarily on human monocytes and macrophages and exists in both membrane-bound and soluble forms. Bacterial products translocated from the gut into the systemic circulation such as lipopolysaccharide (LPS) can activate CD14 and result in the release of proinflammatory cytokines.^18,19^ Once stimulated by LPS, CD14 can shed from monocytes and macrophages. It can then activate cells that usually lack CD14 and render them sensitive to activation by LPS. Therefore, via this pathway, even low levels of LPS can initiate a cytokine cascade.^20^ The resultant cytokine cascade includes the proinflammatory cytokines tumor necrosis factor (TNF)-α, IL-1β, and IL-6, which have been shown to be associated with poor neurodevelopment.^6^ However, the association between sCD14 and neurodevelopmental outcomes remains unknown.

The goal of this study is twofold: 1) to validate our previous findings of inflammatory markers in an independent cohort and 2) to test the hypothesis of a link between EE and neurodevelopment. To these ends, we aimed to determine whether elevated levels of sCD14 would be associated with lower neurodevelopmental scores. We also measured downstream inflammatory mediators from CD14 activation including the proinflammatory cytokines TNF-α, IL-1β, IL-6, and C-reactive protein (CRP). We hypothesized that elevated levels of proinflammatory cytokines, sCD14, and CRP would be associated with lower neurodevelopmental scores.

## METHODS

### Study population and enrollment.

We conducted a longitudinal analysis of 422 infants from a cohort of 700 children from the PROVIDE study, an ongoing community-based prospective cohort study of enteric infections and oral polio vaccine outcomes in infants from a slum of Mirpur in Dhaka, Bangladesh. The study design and recruitment of the PROVIDE cohort has been extensively described previously.^21^ In brief, 700 infants and their mothers were followed from birth by twice weekly household visits and scheduled clinic visits for the first 3 years of life. There was rolling admission of subjects over the first 18 months and the study period reported here spanned from May 2011 to November 2014. The study was approved by the Ethical Review Board of the International Centre for Diarrhoeal Disease Research, Bangladesh, as well as by the Institutional Review Boards of the University of Virginia and the University of Vermont.

### Anthropometrics.

Anthropometric measurements of each child were obtained at enrollment and at the time of neurodevelopmental testing at 78 and 104 weeks. The length of each child was measured to the nearest 0.1 cm (Infantometer Baby Board, Seca 416). Each child was weighed in light clothing on an electronic scale (Digital Baby and Toddler Scales, Seca 354), and the weight was recorded to the nearest 0.01 kg. Length and weight measurements were taken twice, and the average of the two measurements was recorded. Anthropometric measurements were converted to length-for-age Z-score (LAZ) and weight-for-age Z-score (WAZ) using WHO Anthro software, version 3.0.1 (Geneva, Switzerland).

### Biomarker measurement.

Sera from infants at 18 weeks of age were tested for IL-1β, IL-6, TNF-α, IL-4, and IL-10 using Human Bio-Plex Pro Assays (*N* = 422). sCD14 was also measured in infant sera at 18 weeks of age using enzyme-linked immunosorbent assay (ELISA) (R and D Systems, Minneapolis, MN). CRP was measured in sera at 6, 18, 40, 53, and 104 weeks using an ELISA (Immundiagnostik AG).

### Neurodevelopment measurement.

Trained child psychologists, blinded to the children’s histories and clinical parameters, assessed cognitive, language, motor, and social emotional development in a clinic setting using a culturally adapted version of the Bayley-III at 78 (*N* = 205) and 104 weeks (*N* = 422). The Bayley-III raw scores for cognitive, language, motor, and social emotional development were converted to norm-referenced standardized scores (mean = 100, standard deviation [SD] = 15) for composite scales. Ten percent of all tests (*N* = 35) were observed by the supervisor (FT) throughout the study period for ongoing reliability. The Bayley Scales have been used by the same research group in several previous studies in rural and urban settings in Bangladesh. Field testing of the instrument showed positive correlations of the Bayley scores with child nutritional status and parental education (*P* < 0.05). Assessment of short-term test–retest reliability (within 7 days) indicated high correlation (*r* > 0.80). Interobserver reliability (intraclass correlation) between tester and trainer was high (*r* = 0.99).^6^

### Maternal depression.

Maternal depression was measured using the Edinburgh Postnatal Depression Scale (EPDS). The EPDS has been validated for use in the postnatal period^22^ and has also been validated in Bangladesh.^23^ The EPDS is a 10-item scale that results in a score ranging from 0 to 30. While the scale alone cannot confirm a diagnosis of depression, a score 10 and above is indicative of the presence of a depressive disorder.^24^

### Statistical analysis.

Infants’ characteristics and risk factors were summarized as counts and percentages for categorical variables and as mean ±SD for continuous variables. For the neurodevelopmental outcomes, we assessed associations of inflammatory markers with the outcomes using linear regression analysis. We first evaluated the effect of baseline variables and inflammatory markers at 78 and 104 weeks on Bayley-III scores using univariate analysis. Based on the results of the univariate analysis, we further performed multivariable analysis to evaluate the associations of biological markers of inflammation with neurodevelopmental outcomes after adjusting for baseline characteristics or potential confounders. As many biomarkers are correlated, such as LAZ and WAZ, we chose only one representative variable from each category we wished to control for. Final multivariable analyses were adjusted for potential confounders of sex, family income, maternal education, and child’s anthropometric status at the time of neurodevelopmental testing.

Because of their skewed distributions, cytokine concentrations values were dichotomized into the highest quartile and lower three quartiles. In our final models, the cytokine measures were analyzed as binary variables (top quartile versus lower three quartiles) as we were most interested in the contribution of elevated levels of cytokines on neurodevelopment. To examine the effect of elevated CRP levels across several time points, an elevated CRP was defined as a value greater than the median CRP value at any particular time point. Statistical significance was defined as a *P* value of < 0.05 (two-tailed). Data were analyzed using IBM SPSS 20 (SPSS Inc., Chicago, IL).

## RESULTS

### Description of the study population.

The Bayley Scales were measured in 205 children at 78 weeks and 422 children at 104 weeks. The Bayley test was administrated to the cohort during an extension phase, and thus 217 children missed the neurodevelopmental measurement at 78 weeks because they were already older than 78 weeks. More than half of children (51.7%) were boys. The children, on average, were malnourished, living in impoverished conditions, and experienced recurrent infection during childhood (Table 1). The children tested at 78 and 104 weeks with the Bayley-III were comparable in their baseline characteristics. The mean household income was < 13,500 taka (BDT) per month (< $174 USD), and about 30% of mothers had no formal education. Average LAZ and WAZ scores declined over the following 104 weeks. At birth, 10% of children were stunted (LAZ < −2); by 104 weeks of age, nearly 40% of children were stunted (Table 1). The mean cognitive, language, motor, and social emotional composite scores at 78 weeks of age were 95.1 ± 6.6 (mean ± SD), 97.0 ± 8.3, 97.2 ± 6.2, and 88.8 ± 6.2, respectively. The mean cognitive, language, motor, and social emotional composite scores at 104 weeks of age were 90.5 ± 5.7, 98.5 ± 8.4, 94.9 ± 7.4, and 94.3 ± 5.6, respectively.

**Table 1:** Descriptive characteristics of the study population of the total cohort at 78 and 104 weeks

Characteristic	78 weeks (*N* = 205)	104 weeks (*N* = 422)
Male sex (%)	106 (51.7)	218 (51.7)
No maternal education (%)	64 (31.2)	124 (29.4)
Monthly family income (BDT)	13264 ± 10183	13408 ± 10101
Maternal BMI < 18.5 (%)	35 (17.1)	74 (17.5)
Edinburgh Postnatal Depression*	108 (55.1)	200 (48.2)
Exclusive breast feeding (days)	98.2 ± 42.7	97.4 ± 41.7
LAZ at birth	−0.91 ± 0.81	−0.94 ± 0.84
Diarrheal illness (total days)†	4.9 ± 6.9	5.6 ± 7.6

ARI = acute respiratory infection; BDT = Bangladeshi taka (currency); BMI = body mass index; LAZ = length-for-age Z-score; SD = standard deviation. Data are expressed as count (%) for categorical measures and mean ± SD for continuous measures.

* Scale > 10 (%).

† During first year of life.

### Malnutrition, socioeconomic status, maternal factors, and cytokines.

Univariate linear regression analyses showed that malnutrition, low socioeconomic status, low maternal education, and maternal depression were significantly and adversely associated with Bayley-III scores at both time points (data not shown). Of note, linear regression showed that elevated levels of IL-1β, IL-6, IL-10, and TNF-α were significantly associated with lower language, and elevated IL-1β also to lower cognitive, language, and social emotional scores at 78 but not 104 weeks (data not shown).

### Soluble CD14 and neurodevelopmental outcomes.

**Figure 1.**
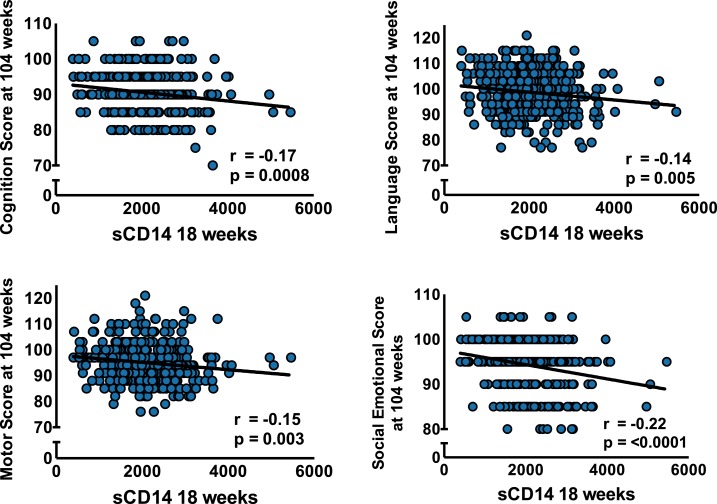
Increased soluble CD14 levels in sera at 18 weeks of age are associated with decreased developmental scores at 104 weeks of age.

sCD14 was associated with lower Bayley-III outcomes across domains (cognition and motor at 78 weeks [data not shown]; all domains at week 104) (Figure 1). The association of sCD14 with Bayley outcomes remained significant in multivariable analysis after controlling for sex, maternal education, monthly family income, and stunting at the time of testing (Table 2).

**Table 2:** Effect of sCD14 on developmental outcomes at 104 weeks adjusting for sex, monthly family income, maternal education, and LAZ at 104 weeks

Predictors	Cognitive Composite Score		Language Composite Score		Motor Composite Score		Social Emotional Composite Score	
	Estimate (SE)*	*P* value	Estimate (SE)*	*P* value	Estimate (SE)*	*P* value	Estimate (SE)*	*P* value
Male sex	0.02 (0.55)	0.705	0.14 (0.81)	**0.005**	−0.02 (0.72)	0.730	0.13 (0.52)	**0.006**
Monthly family income	0.15 (0.00)	**0.003**	0.11 (0.00)	**0.031**	0.03 (0.00)	0.557	0.15 (0.00)	**0.003**
Maternal education (years)	−0.08 (0.61)	0.092	−0.11 (0.90)	**0.024**	−0.07 (0.81)	0.175	−0.83 (0.58)	0.087
LAZ at 104 weeks (every unit)	0.19 (0.29)	**< 0.001**	0.16 (0.43)	**0.002**	0.15 (0.38)	**0.004**	0.21 (0.27)	**< 0.001**
sCD14 at 18 weeks	−0.13 (0.37)	**0.008**	−0.11 (0.001)	**0.022**	−0.13 (0.00)	**0.011**	−0.19 (0.00)	**< 0.001**

LAZ = length-for-age Z-score; sCD14 = soluble CD14; SE = standard error. Bolded values represent a *P* value < 0.05.

* Estimated effect of male sex, monthly family income, maternal education, LAZ at 104 weeks, and sCD14 at 18 weeks on developmental outcomes expressed as the magnitude change in developmental scores for every taka in family income, year of maternal education, and increment in LAZ at 104 weeks. Data in parentheses are SEs for the corresponding covariate effect.

### CRP and neurodevelopmental outcomes.

**Figure 2.**
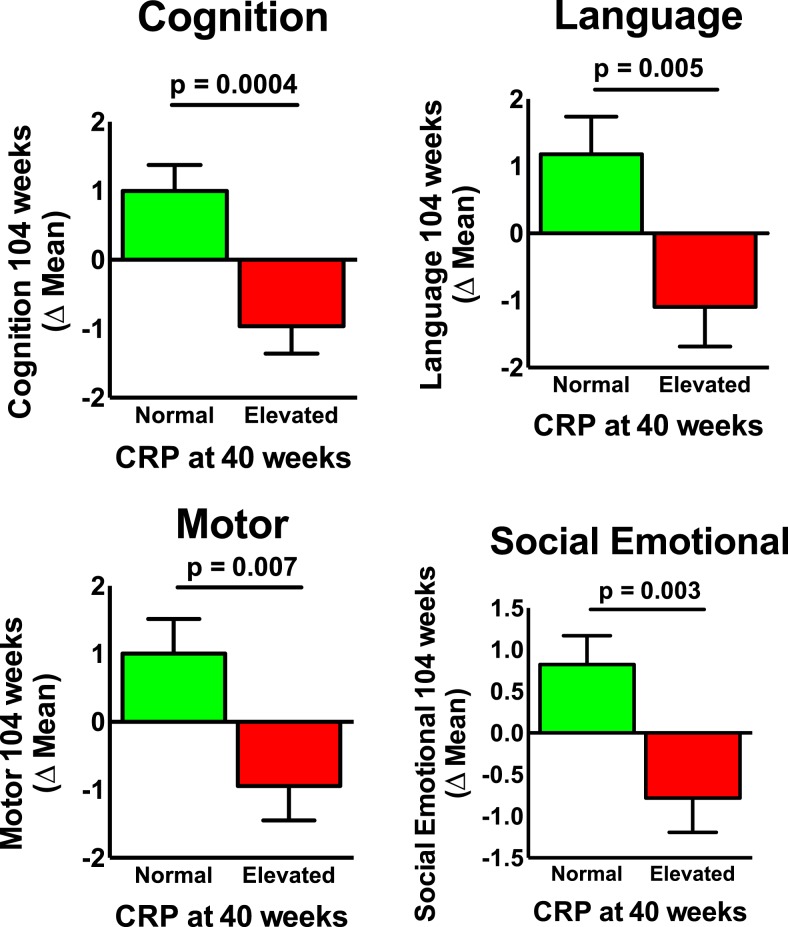
Elevated levels of C-reactive protein at 40 weeks of life are associated with lower developmental scores at 104 weeks of life.

The cumulative number of CRP elevation at 6, 18, 40, 53, and 104 weeks of age was used as a measure of sustained inflammation. We found that elevated CRPs (defined as a value above the median for the age of the child) at 40 and 53 weeks, but not 104 weeks of age, were associated with lower Bayley-III outcomes. In addition, the cumulative number of CRP elevations that a child experienced was also associated with lower Bayley-III results. Figure 2 shows the significant differences in developmental scores between children with normal CRP and those with elevated CRP at 104 weeks. The association of elevated CRP at 40 weeks with developmental scores at 104 weeks remained significant in multivariable analysis after controlling for the same confounders as described earlier (Table 3).

**Table 3:** Effect of elevated CRP on developmental outcomes at 104 weeks adjusting for sex, monthly family income, maternal education, and LAZ at 104 weeks

Predictors	Cognitive Composite Score		Language Composite Score		Motor Composite Score		Social Emotional Composite Score	
	Estimate (SE)*	*P* value	Estimate (SE)*	*P* value	Estimate (SE)*	*P* value	Estimate (SE)*	*P* value
Male sex	0.03 (0.54)	0.474	0.15 (0.80)	**0.002**	−0.01 (0.72)	0.897	0.15 (0.52)	**0.002**
Monthly family income (every taka)	0.16 (0.00)	**0.002**	0.12 (0.00)	**0.021**	0.04 (0.00)	0.454	0.16 (0.00)	**0.001**
Maternal education (years)	−0.08 (0.61)	0.104	−0.12 (0.90)	**0.018**	−0.06 (0.81)	0.212	−0.10 (0.58)	0.050
LAZ at 104 weeks (every unit)	0.18 (0.28)	**< 0.001**	0.15 (0.42)	**0.003**	0.17 (0.37)	**0.001**	0.21 (0.27)	**< 0.001**
Elevated CRP at 40 weeks	−0.15 (0.54)	**0.002**	−0.10 (0.81)	**0.035**	−0.11 (0.73)	**0.033**	−0.12 (0.52)	**0.012**

CRP = C-reactive protein; LAZ = length-for-age Z-score; SE = standard error. Bolded values represent a *P* value < 0.05.

* Estimated effect of male sex, monthly family income, maternal education, LAZ at 104 weeks, and CRP at 40 weeks on developmental outcomes expressed as the magnitude change in developmental scores for every taka in family income, year of maternal education, and increment in LAZ at 104 weeks. Data in parentheses are SEs for the corresponding covariate effect.

## DISCUSSION

The most significant contribution of this study is the validation in an independent cohort of our earlier finding that early life inflammation is associated with lower neurodevelopmental outcomes in a cohort of children growing up in profound adversity.^6^ We found that higher levels of sCD14 in sera was significantly associated with lower neurodevelopmental outcomes in all four domains tested. We also found in this study that IL-1β was significantly associated with lower cognitive, language, and social emotional development. Similarly, elevated levels of the acute phase reactant CRP were associated with lower developmental outcomes in all domains.

The discovery that sCD14 can serve as a marker of neurodevelopment has several important implications. First, our findings of an association of both sCD14 and downstream markers (IL-1β and IL-6)^20^ suggests a common pathway of associated inflammation. Together, these findings are all consistent with the notion that systemic inflammation has a deleterious effect on the developing brain.

Second, the association of sCD14 with neurodevelopmental outcomes may shed light on biological mechanisms that link inflammation with the brain. Although it has been long appreciated that adversity has a detrimental impact on the developing brain, mechanisms that underlie this association still remain unknown.^25^ The association of sCD14 with developmental outcomes implicates a role of the LPS–CD14 pathway in the pathogenesis of neuronal damage. sCD14 has been used in studies in human immunodeficiency virus patients as a marker of microbial translocation, indicating gut barrier dysfunction.^16,26^ The association of sCD14 with developmental outcomes suggests that EE from repeated enteric infections may result in the systemic inflammatory response we see as measured by the proinflammatory cytokine IL-1β and the acute-phase reactant CRP. In fact, the children in this study had on average two different enteropathogens detected in their stool at both 6 and 10 weeks of age and EE (defined by abnormal levels of fecal calprotectin, myeloperoxidase, or alpha-1-antitrypsin) in 80%.^11^ These findings therefore may point to a novel mechanism by which adversity can impact the brains of young children via EE and systemic inflammation.

Third, the association of sCD14 with neurodevelopment may have potential therapeutic implications. If activation of CD14 is the beginning of the pathway, targeted interventions at CD14 could potentially be used to limit excessive inflammation via this pathway and improve outcomes in children. In one study, peripheral blood leukocytes from preterm infants were treated with anti-CD14-blocking antibodies and subsequently stimulated with LPS. CD14 blockade resulted in a significant decrease in the proinflammatory cytokine IL-8.^27^

Our study has several key limitations that should be noted. The Bayley-III Scale has not been normalized for the Bangladesh population, so it is not possible to characterize the results as normal or abnormal. However, the Bayley Scale allowed for internally consistent comparison of children within the cohort. A second limitation was that sCD14, IL-1β, and IL-6 were measured at only 18 weeks, leaving open the possibility that other adversities between 18 weeks and the measurement of Bayley Scales at 78 and 104 weeks of age. We, however, did measure levels of CRP over multiple time points that were associated with neurodevelopmental scores at several time points. Finally, as inflammatory cytokines may be activated through other pathways than LPS–sCD14, our study provides only one possible hypothesis for the mechanism by which systemic inflammation is induced in children who grow up in adversity.

In summary, the present study highlights inflammation as an independent risk factor for impaired infant development. To the best of our knowledge, this is the first study to correlate levels of sCD14 in sera of children with neurodevelopmental outcomes later in life. The causal relationship of the activation of CD14 and subsequent release of downstream inflammatory markers needs to be further investigated. An increased understanding of the interactions between gut inflammation, cytokine levels, and neurocognitive function could allow us to identify at-risk children early for targeted interventions, to allow every child to meet their full developmental potential.
